# Spread of avian pathogenic *Escherichia coli* ST117 O78:H4 in Nordic broiler production

**DOI:** 10.1186/s12864-016-3415-6

**Published:** 2017-01-03

**Authors:** Troels Ronco, Marc Stegger, Rikke Heidemann Olsen, Camilla Sekse, Anne Bang Nordstoga, Tarja Pohjanvirta, Berit Lilje, Ulrike Lyhs, Paal Skytt Andersen, Karl Pedersen

**Affiliations:** 1 National Veterinary Institute, Technical University of Denmark, Bülowsvej 27, 1870 Frederiksberg C, Denmark; 2 Statens Serum Institut, Department of Microbiology and Infection Control, Artillerivej 5, 2300 Copenhagen S, Denmark; 3 Department of Veterinary Disease Biology, University of Copenhagen, Stigbøjlen 4, 1870 Frederiksberg C, Denmark; 4 Norwegian Veterinary Institute, Ullevaalsveien 68, 0454 Oslo, Norway; 5 Finnish Food Safety Authority, Veterinary Bacteriology, Neulaniementie 4, FI-70210 Kuopio, Finland

**Keywords:** APEC, Colibacillosis, Comparative genomics, Phylogenetic analysis, Virulence factors

## Abstract

**Background:**

*Escherichia coli* infections known as colibacillosis constitute a considerable challenge to poultry farmers worldwide, in terms of decreased animal welfare and production economy. Colibacillosis is caused by avian pathogenic *E. coli* (APEC). APEC strains are extraintestinal pathogenic *E. coli* and have in general been characterized as being a genetically diverse population. In the Nordic countries, poultry farmers depend on import of Swedish broiler breeders which are part of a breeding pyramid. During 2014 to 2016, an increased occurrence of colibacillosis on Nordic broiler chicken farms was reported. The aim of this study was to investigate the genetic diversity among *E. coli* isolates collected on poultry farms with colibacillosis issues, using whole genome sequencing.

**Methods:**

Hundred and fourteen bacterial isolates from both broilers and broiler breeders were whole genome sequenced. The majority of isolates were collected from poultry with colibacillosis on Nordic farms. Subsequently, comparative genomic analyses were carried out. This included *in silico* typing (sero- and multi-locus sequence typing), identification of virulence and resistance genes and phylogenetic analyses based on single nucleotide polymorphisms.

**Results:**

In general, the characterized poultry isolates constituted a genetically diverse population. However, the phylogenetic analyses revealed a major clade of 47 closely related ST117 O78:H4 isolates. The isolates in this clade were collected from broiler chickens and breeders with colibacillosis in multiple Nordic countries. They clustered together with a human ST117 isolate and all carried virulence genes that previously have been associated with human uropathogenic *E. coli*.

**Conclusions:**

The investigation revealed a lineage of ST117 O78:H4 isolates collected in different Nordic countries from diseased broilers and breeders. The data indicate that the closely related ST117 O78:H4 strains have been transferred vertically through the broiler breeding pyramid into distantly located farms across the Nordic countries.

**Electronic supplementary material:**

The online version of this article (doi:10.1186/s12864-016-3415-6) contains supplementary material, which is available to authorized users.

## Background

*Escherichia coli* infections in poultry constitute a severe animal health issue and a considerable burden to farmers worldwide, in terms of decreased animal welfare and production economy [1, 2]. Disease in poultry caused by avian pathogenic *E. coli* (APEC) may cause a wide range of extraintestinal symptoms, collectively termed colibacillosis. APEC belong to the group of extraintestinal pathogenic *E. coli* (ExPEC) [3–5], that also includes the pathotypes; uropathogenic *E. coli* (UPEC), neonatal-meningitis *E. coli* (NMEC) and septicemic *E. coli*. All groups have been associated with disease in both humans and animals [3, 4], and it has been reported that human ExPEC strains are closely related to APEC strains, suggesting that poultry could constitute a reservoir of zoonotic APEC strains [3, 6, 7].

In general, APEC isolates from chickens constitute a genetically diverse population with numerous of different serogroups and sequence types (STs). The most commonly observed serogroups are O1, O2 and O78 [6–9], and multilocus sequence typing (MLST) has shown that STs 10, 48, 95 and 117 have been frequently observed [5, 10, 11]. Several types of virulence genes are commonly identified in APEC as well as in human ExPEC [7, 12] (Additional file 1: Table S1). These are often carried on virulence plasmids and pathogenicity islands (PAIs) [13–15].

Nordic broiler production depends on a breeding pyramid where Swedish grandparents are mainly imported from Scotland and used for breeding of parents for export to farms in the rest of the Nordic countries [16, 17]. Interestingly, previous studies indicated that extended-spectrum beta-lactamase (ESBL)-producing *E. coli* can be transmitted vertically from parents to offspring through the breeding pyramid [17–19]. Hence, if great grandparents are infected with virulent *E. coli*, they can potentially disseminate vertically to Swedish grandparents and hereafter to parents and broilers across the Nordic countries.

From 2009 until late 2014, the mortality on Danish poultry farms has on average been decreasing. Hereafter, the mortality has increased to >4.0% in 2015 and it has been suggested that colibacillosis in both parents and broilers has played a significant role [20]. In the same period similar problems with colibacillosis and increased mortality have also been observed on Finnish and Norwegian farms (Magne Hansen, Animalia, pers. comm). The aim of this study was to investigate, using whole genome sequencing, the genetic diversity and potential relatedness of APEC isolates associated with increased mortality and colibacillosis in Nordic countries.

## Methods

### *E. coli* isolates

In this study, 107 bacterial isolates from Danish (*n* = 74), Finnish (*n* = 15), Norwegian (*n* = 16) and Polish (*n* = 2) farms were analyzed. Additionally, assembled draft genomes obtained from seven bacterial isolates collected from diseased Danish chickens, were kindly provided by the Danish poultry industry, and their isolation ID have in this study been assigned a capital “A” (Additional file 2: Table S2). In total, the 114 isolates were collected from 88 different farms and if isolates were from the same farm they were in general collected from different houses. The majority of isolates were collected from diseased broiler chickens and parents (layer hens) and diseased birds were diagnosed with a generalized *E. coli* infection, whereas 15 isolates were collected from healthy birds (Additional file 2: Table S2). The Danish isolates were collected from all parts of the country by the two commercial laboratories at LVK (Landbrugets Veterinære Konsulenttjeneste, Hobro, Denmark) and the poultry slaughterhouse, Danpo (Danpo A/S, Aars, Denmark). The Danish farms were not geographically clustered, but distributed evenly throughout the regions with poultry production. Notably, the draft genomes of two Danish isolates (E44 and E51) have previously been annotated and deposited in DDBJ/ENA/GenBank under the accession numbers LXWV00000000 (E44) and LYPJ00000000 (E51) [21], due to their inclusion in a Danish autogenous vaccine program. Finnish isolates were collected from the Southwestern part of Finland by the Finnish Food Safety Authority (Evira). In Norway, the isolates were collected from central, South-eastern and Western parts of the country by the Norwegian Veterinary Institute (NVI). In both Finland and Norway, the samples were collected from regions where most broiler farms are located.

The Illumina reads sequenced in this study were deposited in the NCBI SRA [22] under the study accession number SRP092633.

### DNA purification and sequencing

Isolates were grown overnight at 37 °C on blood agar (Columbia agar base [Oxoid, Hampshire, UK]) supplemented with 5% calf blood [SSI, Copenhagen, DK]). Single colonies were harvested directly from the agar plates and genomic DNA was purified using the QIAamp DNA Mini Kit (Qiagen, Hilden, Germany), according to the manufacturer’s instructions. The DNA libraries were generated using Nextera XT kit (Illumina Inc., San Diego, Ca) according to manufacturer’s instructions. Finally, Illumina’s MiSeq platform was used for paired-end DNA sequencing with a read length of 2 × 251 bp for all isolates except the seven isolates from the Danish poultry industry which were sequenced with a read length of 2 × 300 bp.

### *De novo* assembly and typing

Raw reads were *de novo* assembled using CLC bio’s Genomics Workbench (GW) v6.5 (Qiagen, Aarhus, Denmark) with default setting and a threshold on contigs of minimum 500 nt. Subsequently, the *de novo* assembled contigs were MLST [23] and serotyped [24] *in silico* using online typing tools [25].

### Core genome diversity

The genetic relationship between all 114 isolates was investigated using single nucleotide polymorphisms (SNPs). SNPs were identified using NASP 1.0 [26] by aligning Illumina reads against *E. coli* strain CFT073 (GenBank accession no. AE014075), using the Burrows-Wheeler Aligner (BWA) [27] after removal of duplicated regions in the reference using NUCmer [28]. GATK Unified Genotyper [29] was used to identify variant positions and to remove positions with <90% unambiguous base calls, as well as SNPs in positions that did not meet a minimum coverage requirement of ≥10×. Subsequently, a phylogenetic tree model was constructed using the maximum-likelihood algorithm implemented in PhyML [30] using Smart Model Selection and the Bayesian Information Criterion with 100 bootstrap replicates and visualized using iTOL v3.1 [31].

Further investigations were performed on all identified ST117 isolates from this study (*n* = 62) together with all 21 identified ST117 *E. coli* strains available at [32] (Additional file 3: Table S3). SNPs were identified as described above but with purging of recombinant regions using Gubbins v1.4.4 with standard settings of five iterations. Phylogenetic analyses on the purged dataset were performed as previously described.

### Identification of virulence and resistance genes

Various types of virulence genes were identified in *de novo* assembled contigs using MyDbFinder v﻿1.1 [33] and antibiotic resistance genes were identified using ResFinder v﻿2.1 [34]. Further descriptions and Genbank accession numbers of the selection of virulence genes are found in Additional file 1: Table S1. In some cases, CLC bio’s Genomics workbench was used to verify the presence of the open reading frames by BLASTN and mapping of reference genes to the *de novo* assembled contigs.

## Results

The majority of the isolates (66/114) were sequenced to an average coverage of ≥50 fold, whereas 35/114 of the draft genomes had an average coverage of >30. The rest had an average coverage of >18, whereas seven draft genomes provided by the Danish poultry industry exhibited slightly less coverage. Assembly metrics (average coverage, N50, number of contigs and assembly size) can be found in Additional file 4: Table S4.

### Serotyping and MLST

**Fig. 1:**
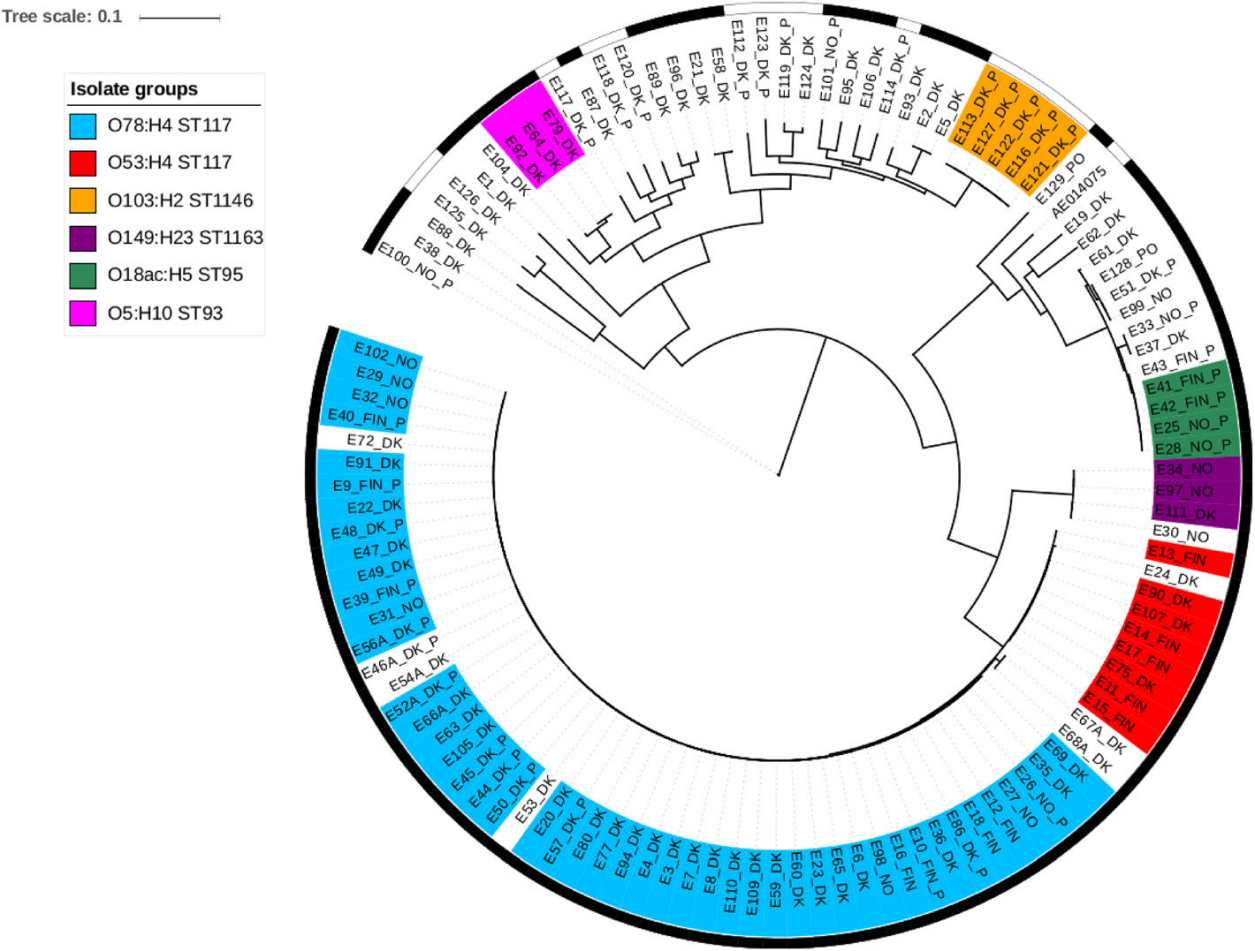
Maximum-likelihood tree of 114 *E. coli* isolates based on 145,637 core SNPs. The analysis shows a clade of 62 APEC isolates collected from both broilers and parents (P) on Danish (DK), Finnish (FIN) and Norwegian (NO) chicken farms. All 62 isolates belonged to ST117. Isolate groups of the same serotype and ST are presented in identical colors. Isolates collected from diseased animals are marked with a black strip, whereas the white strip indicates isolates from healthy chickens. *E. coli* strain CFT073 served as reference and the scale indicates substitutions per site

Serotype genes were in the majority of the 114 assembled genomes, identified with thresholds of ≥90% nucleotide identity, ≥90% coverage of the query and a sequence depth of >10×. However, in eight isolates (E29, E50, E52, E56, E64, E66, E69 and E91) the O-type genes were on average identified with 67% coverage of the query and ≥90% nucleotide identity, whereas no O-﻿type genes were identified in nine isolates (Additional file 2: Table S2). The isolates showed a high diversity with a total of 33 different serotypes, and MLST analyses identified 29 different STs. The most prevalent serotype was O78:H4 observed among 43% (49/114) of the isolates, whereas 54% (62/114) were found to be of ST117 (Additional file 2: Table S2). Of all isolates, 61% (70/114) could be divided into six groups associated with the same serotype and ST (Table 1). Notably, all six groups presented in Table 1 were also closely related according to the SNP analysis (Fig. 1).

**Table 1:** Virulence gene content among 114 *E. coli* isolates

	Group 1	Group 2	Group 3	Group 4	Group 5	Group 6	Healthy	Diseased
Isolates	47	8	5	4	3	3	15	99
Farms	34	8	4	4	2	3	15	73
Serotype	O78:H4	O53:H4	O103:H2	O18ac:H7	O149:H23	O5:H10	-	-
ST	117	117	1146	95	1163	93	-	-
*fimA*	+	+	+	+	+	1/3	12/15	94/99
*fimC*	+	+	+	+	+	+	13/15	96/99
*papC*								4/99
*tsh*				+		+		20/99
*fyuA*		+		3/4			1/15	28/99
*iroD*	+	+		+	+	+	4/15	94/99
*iroN*	+	+		+	+	+	3/15	93/99
*irp2*		+		+			1/15	30/99
*iucA*	+	+	+	+		+	7/15	84/99
*iucD*	+	+	+	+		1/3	7/15	84/99
*cva*		+	+	+	+	+	12/15	39/99
*cvi*		+	+	+	+	+	12/15	39/99
*iss*	+	+	2/5	+	+	+	7/15	97/99
*ompA*	+	+	+	+	+	+	15/15	99/99
*vat*	+	+		+				71/99
*hlyA*								
*ibeA*				+		1/3		13/99

The table shows the virulence gene content among *E coli* groups of identical ST and serotype. “+” indicates presence of genes. If a virulence gene was not present among all isolates in a group, the ratio of isolates that carried the gene is presented. The number of isolates and the number of different farms they were collected from, in each group is shown. The isolates from these groups were also closely related according to the SNP analysis, and the groups are highlighted in colors in Fig. 1. Furthermore, the ratios of isolates from healthy and diseased poultry that carried virulence genes are shown

### Phylogenetic analyses

The SNP calling based on all 114 isolates had a total of 145,637 variant positions identified in ~49% of the reference genome. The phylogenetic analysis revealed a large clade of 62 isolates from both diseased broilers and parents collected in Denmark, Finland and Norway. Isolates from healthy chickens were not related to this clade (Fig. 1). All isolates from this clade belonged to ST117 and 47 of these had serotype O78:H4 whereas eight had O53:H5 (Fig. 1). It was not possible to identify O-type genes in six isolates from this clade, but they all carried the H4 gene. A single isolates (E24) was of serotype O161:H4 (Fig. 1) (Additional file 2: Table S2). Minor clusters of few isolates with identical serotype and ST were present but none of them contained isolates from more than two different countries (Fig. 1).

**Fig. 2:**
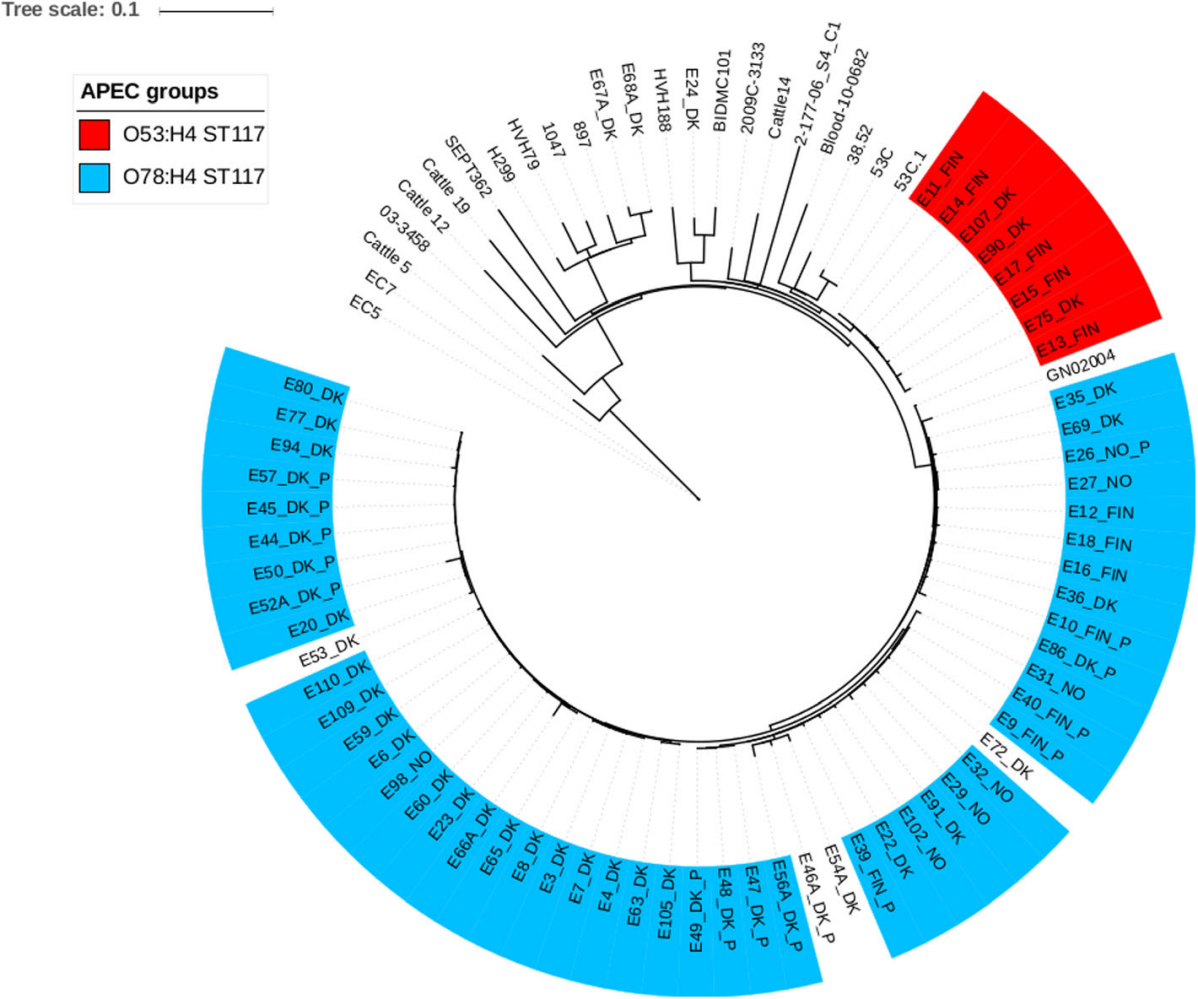
Maximum likelihood tree of 83 ST117 *E. coli* based on 2,617 purged core SNPs. The analysis includes 62 ST117 poultry isolates and 21 international strains. A distinct clade of 53 closely related APEC isolates collected from both broilers and parents (P) on Danish (DK), Finnish (FIN) and Norwegian (NO) chicken farms was identified. Fourty-seven of these isolates were of serotype O78:H4 (blue), whereas a single was O53:H4 (red). All isolates from this study were from diseased birds and the reference strain *E. coli* strain CFT073 is not included. The scale indicates substitutions per site

The SNP calling based on all 83 ST117 genomes from both this study and those obtainable from the public domain at NCBI [32] had a total of 13,215 variant positions identified in ~64% of the genomes, and with 2,617 SNPs remaining after purging of recombinant regions. The analysis identified a major clade primarily consisting of 47 O78:H4 isolates from both diseased broilers and parents collected on 34 different farms in Denmark, Finland and Norway (Fig. 2). On average, the length between these 47 isolates was 23 SNPs. In four isolates (E46, E53, E54, and E72) from this clade no O-type genes were identified, whereas only one isolate in this clade (E13) belonged to a serogroup different from O78 (Fig. 2). Furthermore, strain GN02004 obtained from the RefSeq archive at NCBI (Additional file 3: Table S3) belonged to serotype O24:H4. The 47 O78:H4 isolates differed by at least 50 SNPs from the O53:H4 clade (Fig. 2).

### Identification of virulence and resistance genes

In general, different combinations of virulence genes were identified in the 114 draft genomes with thresholds of ≥90% nucleotide identity and ≥90% coverage of the query sequence. (Table 1 and Additional file 2: Table S2). All 47 ST117 O78:H4 isolates carried a unique combination of nine virulence genes compared to the eight ST117 O53:H4 isolates (Table 1). However, the isolates in this cluster did not carry any of the investigated virulence genes that were not also present in other isolates, i.e., no virulence genes were unique for this cluster. *hlyA* was not identified in any of the isolates whereas *fimA*/*C* and *ompA* were found in almost all isolates. *papC*, *tsh*, *vat* and *ibeA* were only associated with diseased poultry and *iroD*, *iroN* and *iss* were found in ≥94% (93/99) of the isolates from diseased birds (Table 1). The majority (79/114) of all isolates did not carry antibiotic resistance genes. Only few (6/47) of the ST117 O78:H4 isolates carried antibiotic resistance genes, whereas resistance genes were more common among the other groups (Additional file 2: Table S2). The most commonly identified resistance genes were against β-lactams, sulphonamides or streptomycin, and less often against tetracycline or trimethoprim.

## Discussion

In recent years (2014–2016), an increase in cases of colibacillosis on Nordic poultry farms has caused a raise in mortality and economic losses [20] (Magne Hansen, Animalia, pers. comm). Therefore, the genetic diversity among 114 *E. coli* isolates mainly collected from diseased animals on poultry farms with colibacillosis were investigated. In agreement with previous studies [5, 7, 9, 11], it was found that the poultry isolates were a genetically diverse population. However, we identified a group of 47 closely related ST117 O78:H4 isolates collected from diseased broilers and parents in multiple Nordic countries, which shared a similar genetic background (Fig. 2). In concordance, it has previously been reported that APEC isolates are widely associated with serogroup O78 and ST117 [7, 11]. These 47 isolates were not related to any of the isolates from healthy chickens (Fig. 1). On average, the distance between these 47 isolates was 23 SNPs, whereas the distance to the closest related ST117 O53:H4 isolate outside this group was 50 SNPs (Fig. 2). Additionally, all 47 ST117 O78:H4 carried an identical and unique combination of virulence genes compared to all other investigated isolates (Table 1). Thus, according to both the investigation of virulence profiles and the SNP analyses the 47 ST117 O78:H4 isolates define a distinct lineage. The isolates from this lineage carried nine genes that encode virulence factors important in the pathogenesis of avian colibacillosis (Table 1). Interestingly, O78 strains have been suggested to be the main cause of avian colisepticemia together with O1 and O2 strains [35]. The O-antigen capsule allows bacteria to avoid the host’s innate immune response and studies have shown that this LPS capsule is required during systemic infections [35, 36]. Type I fimbriae encoded by *fimA*/*C*, have been shown to be necessary for initial colonization of the respiratory system [37]. Additionally, previous studies suggest that *iucA*/*D* and *iroD* which encode aerobactin and salmochelin siderophores respectively, are specifically important for iron acquisition in the extraintestinal environment of chickens [38]. As in this study, the virulence factor increased serum survival encoded by *iss,* was previously identified in APEC strains [7, 12] as well as the vacuolating autotransporter toxin Vat, but their exact role in the pathogenesis needs to be further elucidated [15, 39]. It should also be noticed that some of the virulence genes (*fimA*/*C*, *ompA*) were found among almost all analyzed isolates, both from healthy and diseased birds, which indicates that they are not only involved in avian colibacillosis. Additionally, a wide range of other virulence factors have also been suggested to be associated with APEC [40]. None of the investigated virulence genes (Table 1) were found exclusively among the O78:H4 cluster and it may therefore be suggested that other, yet undefined virulence mechanisms were partly responsible for the high virulence of this strain. Thus, a comparative study to reveal more precisely why the O78:H4 ST117 lineage was considerably associated with increased mortality among Nordic broilers and breeders from 2014–2016 could be interesting to carry out.

In the Nordic countries, all poultry farms receive their parents from Swedish hatcheries where grandparents are imported from Scotland [16, 17]. It was not possible to verify how many Swedish parent flocks that the birds included in this study originated from, which could have further revealed the extent of the colibacillosis issues observed on Nordic poultry farms. Finding highly similar isolates in broilers and parents from distantly located farms that share one common source for parent animals strongly support a vertical dissemination of ST117 O78:H4 isolates from grandparents and great grandparent. Vertical transmission of pathogenic *E. coli* has previously been observed in other studies [17–19]. Unfortunately, *E. coli* isolates from diseased grandparents or great grandparents were not available to further confirm this hypothesis. It could have been interesting to investigate samples from parents and their corresponding offspring but a parent flock can be origin to several different broiler flocks. Thus, it is not possible to collect samples directly from parent/offspring pairs [16]. Instead, an in vivo infection study of parents and their offspring could be carried out.

The 47 O78:H4 ST117 isolates carried various types of virulence genes (Table 1) that previously have been identified in both APEC and human UPEC isolates. Additionally, they were closely related to the ST117 O24:H4 *E. coli* strain GN02004 from NCBI (Fig. 2), which previously has been collected from human body fluids. (Additional file 3: Table S3). Therefore, it could be speculated whether the origin of these colibacillosis cases could have been the introduction of human UPEC to grandparents or great grandparents in the upper parts of the breeding pyramid. Strain GN02004 was sampled in USA but we could not obtain further information regarding possible dissemination via travel activities to Europe. Thus, diseased chickens could potentially constitute a zoonotic risk to e.g., farmers or other people who get in contact with them [3, 7]. Additionally, *E. coli* strains have been found to end up in the broiler chicken meat [17].

Since the increase in the occurrence of colibacillosis has a considerable impact on animal welfare and production economy, it is of great importance to obtain more in-depth knowledge regarding APEC and colibacillosis and to develop vaccines that possibly could provide immunization of the poultry. Interestingly, *E. coli* strain E44 [21] collected from a diseased parent bird, was here shown to be a part of the major ST117 O78:H4 linage. In 2015, strain E44 was selected for a Danish autovaccine program due to its suggested relation to the increase in colibacillosis on Danish poultry farms. However, the efficiency of the program remains to be evaluated.

## Conclusion

Genomic investigation of APEC isolates collected from diseased chickens on Nordic poultry farms revealed the presence of a predominant lineage of ST117 O78:H4 isolates. The analyses indicated that the ST117 O78:H4 strains have been transmitted vertically through the broiler breeding pyramid and contributed considerably to the increase of colibacillosis cases observed on Nordic poultry farms from 2014 to 2016.

## Additional files

Additional file 1: Table S1. APEC- and human UPEC-associated virulence genes. The pdf file contains descriptions of APEC- and human UPEC-associated virulence genes including GenBank accessions numbers and references. (PDF 295 kb)
Additional file 2: Table S2.
*E. coli* isolates from chicken farms. The pdf file contains information regarding the farm, house, source, country sample date and state of animal associated with each of the 114 poultry isolates. Furthermore, the serotypes and STs are presented together with virulence and resistance gene content. (PDF 806 kb)
Additional file 3: Table S3. ST117 *E. coli* strains from NCBI. The pdf file contains information regarding all 21 international ST117 *E. coli* isolates available at NCBI [32]. The host, collection date, country and isolation source associated with each isolate are included together with the GenBank accession number. (PDF 295 kb)
Additional file 4: Table S4. Assembly metrics for *E. coli* isolates. This pdf file shows assembly metrics (average coverage, N50, number of contigs and assembly size) for the 114 isolates. (PDF 436 kb)
