# Supplementary material for: Spread of avian pathogenic Escherichia coli ST117 O78:H4 in Nordic broiler production
Source: BMC Genomics. 2017 Jan 3;18:13. doi: 10.1186/s12864-016-3415-6 (PMC5210278; doi:10.1186/s12864-016-3415-6)
Supplement: Additional file 1: Table S1. — APEC- and human UPEC-associated virulence genes. The pdf file contains descriptions of APEC- and human UPEC-associated virulence genes including GenBank accessions numbers and references. (PDF 295 kb) [file 12864_2016_3415_MOESM1_ESM.pdf]

Table S1. APEC- and human UPEC-associated virulence genes

Genes	Description	Accession No	Reference
**Adhesins**			
fimC	Type 1 fimbriae	CP004009	[1]
papC	Pilus associated with pyelonephritis	AM690766	[2]
fimA	Type 1 fimbriae	NC_000913	[3]
tsh	Temperature-sensitive haemagglutinin	AF218073	[4]
**Iron acquisition**			
fyuA	Ferric Yersinia uptake	Z38064	[5]
iroN	Catecholate siderophore	DQ381420	[6]
irp2	Iron-repressible protein	L18881	[7]
iucA	Aerobactin	X76100	-
iucD	Aerobactin	M18968	[8]
iroD	Salmochelin siderophore	DQ381420	[6]
**Protectins/serum resistance**			
cva/cvi	colicin V genes	AJ223631	[9]
iss	Increased serum survival	AF042279	[10]
ompA	Outer membrane protein	CP004009	[1]
**Toxins**			
vat	Vacuolating autotransporter toxin	AY151282	[11]
hlyA	Haemolysin A	FM180012	[12]
**Invasins**			
ibeA	Invasion of brain endothelium	CP000468	[13]

Description of APEC- and human UPEC-associated virulence genes [14][15]

References

1. Mangiamele P, Nicholson B, Wannemuehler Y, Seemann T, Logue CM, Li G, et al. Complete genome sequence of the avian pathogenic *Escherichia coli* strain APEC O78. Genome Announc. 2013;1:e0002613.

2. Zdziarski J, Svanborg C, Wullt B, Hacker J, Dobrindt U. Molecular basis of commensalism in the urinary tract: Low virulence or virulence attenuation? Infect. Immun. 2008;76:695–703.

3. Riley M, Abe T, Arnaud MB, Berlyn MKB, Blattner FR, Chaudhuri RR, et al. *Escherichia coli* K-12: a cooperatively developed annotation snapsh–2005. Nucleic Acids Res. 2006;34:1–9.

4. Dozois CM, Dho-Moulin M, Brée A, Fairbrother JM, Desautels C, Curtiss R. Relationship between the Tsh autotransporter and pathogenicity of avian Escherichia coli and localization and analysis of the *tsh* genetic region. Infect. Immun. 2000;68:4145–54.

5. Rakin A, Urbitsch P, Heesemann J. Evidence for two evolutionary lineages of highly pathogenic *Yersinia* species. J. Bacteriol. 1995;177:2292–8.

6. Johnson TJ, Johnson SJ, Nolan LK. Complete DNA sequence of a ColBM plasmid from avian pathogenic *Escherichia coli* suggests that it evolved from closely related ColV virulence plasmids. J. Bacteriol. 2006;188:5975–83.

7. Guilvout I, Mercereau-Puijalon O, Bonnefoy S, Pugsley AP, Carniel E. High-molecular weight protein 2 of *Yersinia enterocolitica* is homologous to AngR of *Vibrio anguillarum* and belongs to a family of proteins involved in non-ribosomal peptide synthesis. J. Bacteriol. 1993;175:5488–504.

8. Herrero M, De Lorenzo V, Neilands JB. Nucleotide sequence of the *iucD* gene of the pCoIV-K30 aerobactin operon and topology of its product studied with *phoA* and *lacZ* gene fusions. J. Bacteriol. 1988;170:56–64.

9. Otto BR, van Dooren SJM, Nuijens JH, Luirink J, Oudega B. Characterization of a hemoglobin protease secreted by the pathogenic *Escherichia coli* strain EB1. J. Exp. Med. 1998;188:1091–103.

10. Chuba PJ, Leon MA, Banerjee A, Palehaudhuri S. Cloning and DNA sequence of plasmid determinant. Mol Gen Genet. 1989;216:287–92.

11. Parreira VR, Gyles CL. A novel pathogenicity island integrated adjacent to the thrW tRNA gene of avian pathogenic *Escherichia coli* encodes a vacuolating autotransporter toxin. Infect. Immun. 2003;71:5087–96.

12. Burgos YK, Pries K, Pestana de Castro AF, Beutin L. Characterization of the alpha-haemolysin determinant from the human enteropathogenic *Escherichia coli* O26 plasmid pEO5. FEMS Microbiol. Lett. 2009;292:194–202.

13. Johnson TJ, Kariyawasam S, Wannemuehler Y, Mangiamele P, Johnson SJ, Doetkott C, et al. The genome sequence of avian pathogenic *Escherichia coli* strain O1:K1:H7 shares strong similarities with human extraintestinal pathogenic *E. coli* Genomes. J. Bacteriol. 2007;189:3228–36.

14. Ewers C, Li G, Wilking H, Kießling S, Alt K, Antáo EM, et al. Avian pathogenic, uropathogenic, and newborn meningitis-causing *Escherichia coli*: How closely related are they? Int. J. Med. Microbiol. 2007;297:163–76.

15. Rodriguez-Siek KE, Giddings CW, Doetkott C, Johnson TJ, Fakhr MK, Nolan LK. Comparison of *Escherichia coli* isolates implicated in human urinary tract infection and avian colibacillosis. Microbiology. 2005;151:2097–110.
