# Supplementary material for: Spread of avian pathogenic Escherichia coli ST117 O78:H4 in Nordic broiler production
Source: BMC Genomics. 2017 Jan 3;18:13. doi: 10.1186/s12864-016-3415-6 (PMC5210278; doi:10.1186/s12864-016-3415-6)
Supplement: Additional file 2: Table S2. — E. coli isolates from chicken farms. The pdf file contains information regarding the farm, house, source, country sample date and state of animal associated with each of the 114 poultry isolates. Furthermore, the serotypes and STs are presented together with virulence and resistance gene content. (PDF 806 kb) [file 12864_2016_3415_MOESM2_ESM.pdf]

Table S2. *E. coli* isolates from chicken farms (see abbreviations below the table)

Name	Farm	House	Sero-type	Virulence genes	Antibiotic resistance genes	MLST	Broiler (B)/ Parrent (P)	State	Source	Coun-try	Sample dates
**E1**	1	1	O45: H19	*tsh, iucD, ompA, fimA, fimC, iroN,iroD iucA,iss*	-	ST371	B	D	Liver	DK	310315
**E2**	1	3	O88:H1	*cva, tsh, iucD, iss, iroN,iroD ompA, iucA, fimC,fimA cvi*	*aad, bla, cat, str, sul, tet, dfr*	ST162	B	D	Liver	DK	310315
**E3**	2	3	O78:H4	*iucD,iss,fimA,ompA,iucA, fimC,iroN,iroD,vat*	-	ST117	B	D	Liver	DK	150415
**E4**	2	2	O78:H4	*vat, iucD, iss, ompA,iroD iroN, iucA, fimC, fimA*	-	ST117	B	D	Liver	DK	150415
**E5**	3	3	O88:H1	*cva,cvi, tsh, iucD, iss, iroN, ompA ,iucA, fimC, fimA,iroD*	*aad, bla, cat, str, sul, tet, dfr*	ST162	B	D	Liver	DK	150415
**E6**	3	3	O78:H4	vat, iucD, iss, iucA,iroD ompA, iroN, fimA, fimC	*bla, sul, tet, dfr*	ST117	B	D	Liver	DK	150415
**E7**	4	Unkn	O78:H4	*vat, iucD, iss, fimC, iroN, iucA,fimA,iroD, iucA*	-	ST117	B	D	Liver	DK	150415
**E8**	4	Unkn	O78:H4	*vat, iucD, iss, iroN, iucA, ompA, fimA, fimC,iroD*	*bla*	ST117	B	D	Liver	DK	150415
**E9**	5	1	O78:H4	*vat, iucD, iss, iroN, iucA, ompA, fimC, fimA,iroD*	-	ST117	P	D	BM	FIN	290115
**E10**	6	1	O78:H4	*iucD, iss, iroD, fimC, fimA, iucA, ompA, iroN,vat*	-	ST117	P	D	BM	FIN	280115
**E11**	7	1	O53:H4	*cva, iucD, irp2,iss,vat, iucA, iroN, fyuA, ompA, fimA, fimC,cvi,iroD*	-	ST117	B	D	BM	FIN	130215
**E12**	8	1	O78:H4	*vat, iucD, iss, iroN, iucA, fimC, fimA, ompA,iroD*	-	ST117	B	D	BM	FIN	060215
**E13**	9	1	O53:H4	*cva, vat, iucD, irp2,iss, iroN, fimC,, fimA, fyuA, iucA, ompA,cvi,iroD*	*str*	ST117	B	D	BM	FIN	040215
**E14**	10	1	O53:H4	*cva, iucD, irp2,iss, vat, fimC, fimA, ompA, iroN, iucA, fyuA,cvi,iroD*	*str*	ST117	B	D	BM	FIN	120215
**E15**	11	1	O53:H4	*cva, iucD, irp2, iss,iroD vat, fimA, iroN iucA,cvi , fyuA, ompA, fimC*	*str*	ST117	B	D	BM	FIN	120215
**E16**	12	1	O78:H4	*vat, iucD, iss, iucA, iroN, fimC, fimA, ompA,iroD*	-	ST117	B	D	BM	FIN	130215
**E17**	13	1	O53:H4	*cva, iucD, irp2,iss, vat, fimC iroN,fyuA,cvi,iroD ompA,iucA,fimA*	*str*	ST117	B	D	BM	FIN	130215
**E18**	14	1	O78:H4	*vat, iucD, iss, iroN,iroD ompA, fimC, fimA, iucA*	-	ST117	B	D	BM	FIN	130215
**E19**	15	1	O50O2: H6	*cva, vat, tsh, iucD, irp2, iss, iroN, ompA, fimA,cvi fimC, ibeA, fyuA, iucA,iroD*	*bla*	ST141	B	D	Liver	DK	130515
**E20**	16	2A	O78:H4	*vat, iucD, iss, fimC, iroN, fimA, ompA, iucA,iroD*	-	ST117	B	D	Liver	DK	130515

Table S2.

**Name**	**Farm**	**House**	**Sero-type**	**Virulence genes**	**Antibiotic resistance genes**	**MLST**	**Broiler (B)/ Parrent (P)**	**State**	**Source**	**Coun-try**	**Sample dates**
**E21**	16	1B	O78:H4	*irp2,iss, ompA, fimC, fimA, fyuA, iroN,iroD,iucD*	*tet*	ST23	B	D	Liver	DK	130515
**E22**	17	1	O78:H4	*iss, vat, iucD,, fimA, fimC, iucA, iroN, ompA,iroD*	-	ST117	B	D	Liver	DK	230615
**E23**	18	1	O78:H4	*iss, iucD, vat, fimC, fimA, ompA, iroN, iucA,iroD*	-	ST117	B	D	Liver	DK	150715
**E24**	19	1	O161: H4	*iss, irp2, vat, iucD, iroN, ompA, fyuA, iucA, fimA, fimC,papC,iroD*	*bla, tet*	ST117	B	D	Liver	DK	180815
**E25**	20	1	O18ac: H7	*irp2, tsh,vat,iroD iucD,cva,cvi,ibeA,iss fyuA, fimC, fimA, iucA, iroN, ompA,*	-	ST95	P	D	Unkn	NO	230915
**E26**	21	Unkn	O78:H4	*iss, vat,iucD, iucA, iroN, fimC, fimA, ompA,iroD*	-	ST117	P	D	Femur	NO	260815
**E27**	22	1	O78:H4	*iss,vat, iucD, iroN, fimC, fimA, ompA, iucA,iroD*	*bla*	ST117	B	D	Spleen	NO	070915
**E28**	21	Unkn	O18ac: H7	*iucD, irp2, cvi, cva, ibeA, fyuA, iroD, iucA, fimC, fimA, ompA,tsh,vat, iroN*	*bla*	ST95	P	D	Spleen	NO	130915
**E29**	23	1	O78:H4	*iss, iucD, vat,, iucA, iroN, ompA, fimC, fimA,iroD*	-	ST117	B	D	Liver	NO	160915
**E30**	24	1	H23	*iss,cva, cvi, iroN, fimC, ompA, fimA,iroD*	-	ST1163	B	D	Unkn	NO	040815
**E31**	25	1	O78:H4	*iucD, vat, iroN, fimC,iss fimA, iucA, ompA,iroD*	*bla*	ST117	B	D	Unkn	NO	060815
**E32**	26	1	O78:H4	*iss, iucD, vat ,fimC, fimA, iroN, ompA, iucA,iroD*	*bla*	ST117	B	D	Unkn	NO	170815
**E33**	27	1	O1:H7	*iroN, iucA, ompA,cva,cvi fyuA,irp2 fimA, fimC, iroD,papC,iucD,vat,iss*	-	ST95	P	D	Unkn	NO	100815
**E34**	28	Unkn	O149: H23	*cva,, cvi, iss, iroN, fimC, ompA,iroD,fimA,iss*	-	ST1163	Unkn	D	Femur	NO	160915
**E35**	29	1	O78:H4	*iss, iucD, vat, iroN, fimC, fimA, iucA, ompA,iroD*	-	ST117	B	D	Liver	DK	230915
**E36**	29	2	O78:H4	*iss, iucD ,vat,iroN, fimC, fimA ompA, iucA,iroD*	-	ST117	B	D	Liver	DK	230915
**E37**	30	1	O1:H7	*iss, irp2, iucD, cvi, cva, vat, ompA,iroN, fyuA,iroD iucA, fimC, fimA,papC*	-	ST95	B	D	Liver	DK	061015
**E38**	30	2	O174: H42	*irp2, iucD, cva, tsh, cvi, iroN, fimA, fimC, iucA, ompA,fyuA,iroD,iss*	*tet*	ST648	B	D	Liver	DK	061015
**E39**	31	1	O78:H4	*iss, iucD, iroN, iucA,fimC, fimA, ompA,vat,iroD*	-	ST117	P	D	BM	FIN	070815
**E40**	32	1	O78:H4	*iss,iucD, vat,,iroN, iucA, ompA, fimC, fimA,iroD*	-	ST117	P	D	BM	FIN	030815

Table S2.

Name	Farm	House	Sero-type	Virulence genes	Antibiotic resistance genes	MLST	Broiler (B)/ Parrent (P)	State	Source	Coun-try	Sample dates
**E41**	33	1	O18ac: H7	*iucD, cva, cvi, fimC, tsh, fimA, iucA iroN, ibeA,iss irp2,iroD ompA,vat,*	-	ST95	P	D	BM	FIN	240615
**E42**	34	1	O18ac: H7	*irp2, cvi, cva, iucD, fimC, fimA, iroN, ompA, fyuA, tsh iucA, ibeA,vat,iroD,iss*	-	ST95	P	D	BM	FIN	280415
**E43**	35	1	O18:H7	*iucD, irp2, cva, cvi, fimC, fimA, fyuA, ompA, ibeA, iucA,tsh,iroN,iroD,iss*	-	ST95	P	D	BM	FIN	260515
**E44**	36	1a	O78:H4	*iss, iucD, vat, iroN, fimC, fimA, ompA, iucA,iroD,*	-	ST117	P	D	Liver	DK	200415
**E45**	36	1b	O78:H4	*iss, iucD, vat, iroN,iroD ompA, iucA, fimA, fimC*	-	ST117	P	D	Liver	DK	200415
**E46A**	36	2a	H4	*iucD, iss, iroN, fimC, ompA, fimA, iroD, iucA,vat*	-	ST117	P	D	Liver	DK	200415
**E47**	36	2b	O78:H4	*iss, vat, iucD, iroD, iroN, ompA, iucA, fimA, fimC*	-	ST117	P	D	Liver	DK	200415
**E48**	36	P1	O78:H4	*iss, iucD, vat,iroD iroN, iucA, ompA, fimA, fimC*	-	ST117	P	D	Liver	DK	200415
**E49**	36	P2	O78:H4	*iss, iucD, vat, iroN, iucA, ompA, fimC, fimA,iroD*	-	ST117	P	D	Liver	DK	200415
**E50**	36	P3	O78:H4	*iss iucD, vat, ompA,iroD iroN, fimA, iucA, fimC,*	-	ST117	P	D	Liver	DK	200415
**E51**	37	3a	O2:H5	*cva, cvi, irp2, iucD, tsh, iroN, ompA, ibeA, fimC, fyuA, fimA, iucA,iroD,iss*	-	ST140	P	D	Liver	DK	210115
**E52A**	37	3b	O78:H4	*vat, iucD, fimC, fimA,iss ompA, iucA, iroN,iroD*	-	ST117	P	D	Liver	DK	210115
**E53**	38	1	H4	*iss, iucD, vat, iroN, fimC, ompA, fimA, iucA,iroD*	-	ST117	B	D	Liver	DK	2015
**E54A**	38	2	H4	*vat, iucD, iss, fimC, fimA, ompA, iucA, iroN,iroD*	-	ST117	B	D	Liver	DK	2015
**E56A**	40	1	O78:H4	*vat, iucD, iroN, iroD ompA,iss iucA, fimA, fimC,*	-	ST117	P	D	Liver	DK	170415
**E57**	40	2	O78:H4	*iss, vat, iucD, fimC, iucA, fimA, ompA, iroN,iroD*	*aad, bla, sul, dfr*	ST117	P	D	Liver	DK,	170415
**E58**	41	1	O78:H4	*tsh, irp2, cvi, cva,iss iucD, fimA, iucA,fyuA, fimC iroN,iroD, ompA,*	*Aad, sul, tet*	ST23	B	D	Liver	DK	300415
**E59**	42	1	O78:H4	*iss, iucD, vat, iroN, fimC, fimA, ompA, iucA,iroD*	-	ST117	B	D	Liver	DK	100515

Table S2.

Name	Farm	House	Sero-type	Virulence genes	Antibiotic resistance genes	MLST	Broiler (B)/ Parrent (P)	State	Source	Coun-try	Sample dates
**E60**	43	1	O78:H4	*iss, iucD, vat, iroN,iroD ompA, iucA, fimA, fimC*	-	ST117	B	D	Liver	DK	280415
**E61**	44	2	O50/O2 :H5	*cva,cvi,irp2.tsh,papC iroN,iucA,fyuA,ibeA, fimC,fimA,ompA,vat, iss,iroD*	-	ST95	B	D	Liver	DK	090515
**E62**	44	1	O50/O2 :H5	*cva, vat, iucD, irp2, cvi,, ibeA, fimC, fimA, iucA, ompA, fyuA, iroN,iroD*	-	ST355	B	D	Liver	DK	090515
**E63**	45	1	O78:H4	*iss, vat iucD, fimA, iucA, iroN, ompA, fimC,iroD*	-	ST117	B	D	Liver	DK	280415
**E64**	46	2	H10	*cva, iss, iucD, iroN,iroD, ompA,iucA tsh, cvi*	-	ST93	B	D	Liver	DK	210415
**E65**	47	1	O78:H4	*iss, iucD, vat, iucA, iroN, fimC, fimA, ompA,iroD*	-	ST117	B	D	Liver	DK	120515
**E66A**	47	2	O78:H4	*vat, iucD, fimC, fimA,,iss iroN, ompA, iucA,iroD*	-	ST117	B	D	Liver	DK	120515
**E67A**	48	1	H4	*iss, irp2, iucD, vat,, iroN, fyuA, fimC, fimA, iucA, ompA,iroD*	*aad, bla, str, sul, tet, dfr*	ST117	B	D	Liver	DK	280415
**E68A**	48	2	H4	*iss, irp2, iucD, vat,, iroN, ompA, fyuA, iucA, fimA, fimC,iroD*	*aad, bla, str, sul, tet, dfr*	ST117	B	D	Liver	DK	280415
**E69**	49	Unkn	O78:H4	*iucD,iss,fimC,iroN, iroD,vat, iucA, ompA,fimA*	-	ST117	Unkn	D	PT	DK	171115
**E72**	50	Unkn	H4	*cva,cvi,fimA,fimC,iucA iroD,irp2,iss,iucD, iroN, ompA,vat,iss*	-	ST117	Unkn	D	PC	DK	171115
**E75**	51	Unkn	O53:H4	*cva,cvi,iroD,iss,irp2, iucD,iroN,fimC,fimA, ompA,fyuA,iucA,vat*	*str*	ST117	Unkn	D	Liver	DK	231115
**E77**	52	Unkn	O78:H4	*iucD,iss,iucA,fimA, iroD,vat,iroN,ompA, fimC*	-	ST117	Unkn	D	Femur	DK	301115
**E79**	53	Unkn	O5:H10	*cva,cvi,ompA,fimA, iroD,iss,iucD,iroN, iucA,tsh,ibeA*	-	ST93	Unkn	D	PT	DK	281015
**E80**	54	Unkn	O78:H4	*iucD,iss,ompA,iroN, iucA,vat,fimA,fimC, iroD*	-	ST117	Unkn	D	Liver	DK	261115
**E86**	55	Unkn	O78:H4	*iucD,iss,vat,iroN,iroD, ompA,fimC,fimA,iucA*	-	ST117	P	D	Liver	DK	090116
**E87**	56	1	O53: H18	*cvi,iss,cva, iss,ompA,iroN,fimC, iroD*	*aad bla, sul, tet, dfr*	ST1638	B	D	Liver	DK	070116
**E88**	57	Unkn	H9	*fimA,fimC,ompA*	-	ST38	B	D	Liver	DK	201215
**E89**	43	Unkn	H10	*cva,cvi,irp2,fyuA, ompA,fimC,fimA,iss*	*aad*	ST10	B	D	Liver	DK	111215

Table S2.

Name	Farm	House	Sero-type	Virulence genes	Antibiotic resistance genes	MLST	Broiler (B)/ Parrent (P)	State	Source	Coun-try	Sample dates
**E90**	58	Unkn	O53:H4	*cva,cvi,iss,irp2,iucD, iroN,ompA,fyuA,iucA, fimA,fimC,vat,iroD*	*str*	ST117	B	D	Liver	DK	110116
**E91**	59	Unkn	O78:H4	*iucD,iss,iroD,fimA iucA,vat,iroN,fimC, ompA*	-	ST117	B	D	Liver	DK	041215
**E92**	60	Unkn	O5:H10	*cva,cvi, iss,iucD,iroD, iroN,iucD,ompA,tsh*	-	ST93	B	D	Liver	DK	071215
**E93**	57	Unkn	O18:H7	*cva,cvi,iroD,iss, iroN,fimA,fimC,ompA*	-	Unkn	B	D	Liver	DK	111215
**E94**	61	Unkn	O78:H4	*iucD,iss,iroD vat,iroN,ompA,iucA, fimA,fimC*	-	ST117	B	D	Liver	DK	151215
**E95**	62	Unkn	O78: H49	*fimA,fimC,ompA,iss*	-	ST2248	Unkn	D	Liver	DK	1. quarter 2016
**E96**	63	Unkn	O113: H48	*fimC,ompA,iss*	*str, aad, bla, sul, tet, dfr*	ST10	Unkn	D	Liver	DK	1. quarter 2016
**E97**	28	Unkn	O149: H23	*cva,cvi,iroN,ompA, fimC,fimA,iroD,iss*	-	ST1163	B	D	Femur	NO	160915
**E98**	64	Unkn	O78:H4	*iucD,iss,iroD vat,iroN,ompA,iucA, fimA,fimC*	-	ST117	B	D	Femur	NO	161015
**E99**	65	Unkn	O50/O2 :H5	*cva,cvi,iroD,iss irp2,iucD,tsh,iroN,ibe A,fyuA,ompA,iucA,fim A,fimC*	-	ST140	B	D	PC	NO	231115
**E100**	66	Unkn	O1:H42	*cva,cvi, ,iucA tsh,iss irp2,iucD,fimC, fimA, ,ompA,fyuA,iroD,iroN*	*bla*	ST648	P	D	Unkn	NO	261015
**E101**	67	Unkn	O103: H21	*cva, cvi,iroD,iss, iucD,iroN,iucA,ompA,f imc,fimA,tsh*	-	ST101	P	D	Liver	NO	250115
**E102**	68	Unkn	O78:H4	*iucD,iss,iroD, vat,fimC,fimA,iroN, ompA,iucA*	-	ST117	B	D	Unkn	NO	270915
**E104**	69	Unkn	O75: H42	*ompA,fimC,fimA*	*str*	ST2223	B	D	BM	DK	2015
**E105**	70	Unkn	O78:H4	*iucD,iss,iroD vat,iucA,iroN,fimC, fimA,ompA*	-	ST117	B	D	BM	DK	2015
**E106**	71	Unkn	O18: H49	*cva,cvi,iroD iss,iroN,ompA,fimC, fimA*	-	ST212	B	D	BM	DK	2015
**E107**	72	3	O53: H4	*cva,cvi,iroD iss,irp2,iucD,iroN, ompA,.fyuA,iucA,fimA, fimC,vat*	*dfr, sul, str, aad*	ST117	B	D	BM	DK	2015
**E109**	Unkn	3	O78:H4	*iucD,iss,iroD vat,ompA,iroN,iucA, fimA,fimC*	-	ST117	B	D	BM	DK	2015

Table S2.

Name	Farm	House	Sero-type	Virulence genes	Antibiotic resistance genes	MLST	Broiler (B)/ Parrent (P)	State	Source	Coun-try	Sample dates
**E110**	43	2	O78:H4	*iucD,iss,iroD vat,iroN,fimC,fimA,iucA,ompA*	-	ST117	B	D	BM	DK	2015
**E111**	73	2	O149:H23	*cva,,cvi,iroN,fimC,ompA,iroD,iss*	*aad, bla, sul, dfr*	ST1163	B	D	BM	DK	2015
**E112**	74	3	O8:H8	*cva,cvi,iss,fimA,fimC ompA,iroD,iroN*	-	ST109	P	H	Cloaca	DK	July 2015
**E113**	75	1	O103:H2	*cva,cvi,iucD,ompA,iucA,fimC,fimA,iss*	*sul, bla*	ST1146	P	H	Cloaca	DK	July 2015
**E114**	76	1	O76:H19	*fimA,ompA,fimC*	-	ST675	P	H	Cloaca	DK	July 2015
**E116**	77	1	O103:H2	*cva,cvi,iucD,iucA,fimA fimC,ompA*	*sul, bla*	ST1146	P	H	Cloaca	DK	July 2015
**E117**	78	1	O82:H10	*ompA,fimA,fimC,iss*	-	ST5625	P	H	Cloaca	DK	July 2015
**E118**	79	1	O149:H10	*cva,cvi,iss,iroN,iroD,ompA,fimC,,iss*	-	ST746	P	H	Cloaca	DK	July 2015
**E119**	80	1	O28ac/O42:H21	*cva,cvi,fimA,ompA,fimC*	-	ST3714	P	H	Cloaca	DK	July 2015
**E120**	81	Unkn	O69:H11	*ompA*	-	ST10	P	H	Cloaca	DK	July 2015
**E121**	39	Unkn	O103:H2	*Cva,cvi,iucD,fimA,fimC,iucA,ompA*	*sul, bla*	ST1146	P	H	Cloaca	DK	July 2015
**E122**	82	Unkn	O103:H2	*cva,cvi,iucD,fimA,fimC,iucA,ompA*	*sul, bla*	ST1146	P	H	Cloaca	DK	July 2015
**E123**	83	Unkn	O9:H21	*cva,cvi,fimC,fimA,ompA*	-	ST1642	P	H	Cloaca	DK	July 2015
**E124**	84	Unkn	O8:H20	*cva,cvi,iucD,iucA,ompA*	-	ST155	B	H	Cloaca	DK	100815
**E125**	85	Unkn	O17/O77:H18	*iroN,ompA,fimA,fimC,iucD,cva,cvi,iss*	-	ST69	B	H	Cloaca	DK	100815
**E126**	86	Unkn	O15:H18	*cva,cvi,irp2,iss,iucD,fimC,fimA,iucA,ompA,fyuA,iroN,iroD*	-	ST69	B	H	Cloaca	DK	170815
**E127**	87	Unkn	O103:H2	*cva,cvi,iucD,ompA,fimA,fimC,iucA,iss*	*sul, bla*	ST1146	B	H	Cloaca	DK	100815
**E128**	Unkn	Unkn	O50/O2:H5	*cva,cvi,iss,iucD,irp2,fyuA,ompA,iroN,ibeA,iucA,fimC,fimA,tsh,iroD*	-	ST140	B	D	Liver	PO	1. quarter 2016
**E129**	Unkn	Unkn	O120:H4	*cva,cvi,iss,vat,irp2,ompA,fyuA,iroN,ibeA,iroD,fimA,fimC,iucD*	*sul*	ST428	B	D	Liver	PO	1. quarter 2016

Abbreviations

B: Broiler chickens, BM: Bone Marrow

P: Parents (layer hens) PT: Peritoneum

D: Diseased PC: Pericardia

H: Healthy Unkn: Unknown
