# Supplementary material for: Spread of avian pathogenic Escherichia coli ST117 O78:H4 in Nordic broiler production
Source: BMC Genomics. 2017 Jan 3;18:13. doi: 10.1186/s12864-016-3415-6 (PMC5210278; doi:10.1186/s12864-016-3415-6)
Supplement: Additional file 3: Table S3. — ST117 E. coli strains from NCBI. The pdf file contains information regarding all 21 international ST117 E. coli isolates available at NCBI [32]. The host, collection date, country and isolation source associated with each isolate are included together with the GenBank accession number. (PDF 295 kb) [file 12864_2016_3415_MOESM3_ESM.pdf]

Table S3. ST117 *E. coli* strains from NCBI

Strain	Host	Collection date	Country	Isolation source	GenBank Acc. no.
**H299**	-	-	-	-	NZ_GL884490.1
**SEPT362**	Chicken	Mar-1994	Brazil	Liver	NZ_AOGL00000000
**HVH 79**	Human	2003	Denmark	Blood	NZ_KE699539.1
**HVH 188**	Human	2003	Denmark	Blood	NZ_KE700731.1
**897**	-	-	-	-	NZ_AYQF00000000
**53C**	Chicken	May-2010	Netherlands	Retail meat	NZ_AYRA00000000
**53C.1**	Chicken	May-2010	Netherlands	Retail meat	NZ_JXMX00000000
**1047**	-	-	-	-	NZ_AYQG00000000
**38.52**	-	-	-	-	NZ_AYQH00000000
**03-3458**	-	-	USA	-	NZ_JHNV00000000
**2-177-06_S4_C1**	Human	Jul-2009	Tanzania	Stool	NZ_JNQG00000000
**Blood-10-0682**	Human	2010	USA	Blood	NZ_JSQL00000000
**EC5**	Chicken	Sep-2013	Malaysia	Lever	NZ_JWKF00000000
**EC7**	Chicken	Sep-2013	Malaysia	Spleen	NZ_JWKG00000000
**BIDMC101**	Human	2014	-	-	NZ_KQ087895.1
**2009C-3133**	Human	-	-	Stool	NZ_CP013025
**GN02004.**	Human	-	USA	Body fluid	NZ_LQRU00000000
**Cattle5**	Cattle	1980	France	Feces	NZ_LVLO00000000
**Cattle12**	Cattle	2011	China	Feces	NZ_LVLV00000000
**Cattle14**	Cattle	2011	China	Feces	NZ_LVLX00000000
**Cattle19**	Cattle	2011	China	Feces	NZ_LVMC00000000
