# Supplementary material for: Spread of avian pathogenic Escherichia coli ST117 O78:H4 in Nordic broiler production
Source: BMC Genomics. 2017 Jan 3;18:13. doi: 10.1186/s12864-016-3415-6 (PMC5210278; doi:10.1186/s12864-016-3415-6)
Supplement: Additional file 4: Table S4. — Assembly metrics for E. coli isolates. This pdf file shows assembly metrics (average coverage, N50, number of contigs and assembly size) for the 114 isolates. (PDF 436 kb) [file 12864_2016_3415_MOESM4_ESM.pdf]

Table S4. Assembly metrics of *E. coli* isolates

Isolate	Average coverage (×)	N50 (Kb)	Number of contigs	Assembly size (Mb)
**E1**	48	95.5	214	5.3
**E2**	100	184.6	151	5.0
**E3**	43	59.9	284	5.1
**E4**	49	99.8	178	5.1
**E5**	18	32.6	356	5.0
**E6**	47	112.0	213	5.2
**E7**	41	74.7	226	5.1
**E8**	65	108.3	181	5.1
**E9**	33	67.9	251	5.2
**E10**	34	77.4	372	5.1
**E11**	35	75.7	188	5.1
**E12**	37	52.3	290	5.2
**E13**	33	46.1	467	5.3
**E14**	30	64.0	256	5.1
**E15**	54	123..0	150	5.1
**E16**	45	87.5	245	5.2
**E17**	52	99.4	152	5.1
**E18**	38	58.3	297	5.1
**E19**	39	128.0	144	5.2
**E20**	44	65.9	261	5.1
**E21**	52	128.3	132	5.0
**E22**	48	112.1	238	5.3
**E23**	46	115.1	174	5.1
**E24**	53	166.2	181	5.2
**E25**	36	107.2	162	5.2
**E26**	53	97.9	182	5.1
**E27**	63	87.3	209	5.1
**E28**	55	162.5	137	5.1
**E29**	66	87.4	233	5.2
**E30**	58	85.5	193	5.0
**E31**	59	72.1	283	5.2
**E32**	65	93.5	193	5.2
**E33**	56	85.8	182	5.0
**E34**	72	110.7	132	5.1
**E35**	114	87.4	235	5.3
**E36**	68	87.8	223	5.2
**E37**	99	188.6	142	5.1
**E38**	51	90.3	235	5.3
**E30**	63	72.2	239	5.2
**E40**	94	97.5	221	5.1

Table S4.

Isolate	Average coverage (×)	N50 (Kb)	Number of contigs	Assembly size (Mb)
**E41**	91	135.4	137	5.1
**E42**	46	56.3	229	5.1
**E43**	75	155.0	119	5.2
**E44**	91	83.8	195	5.1
**E45**	86	120.1	204	5.1
**E46A**	>15	-	354	5.2
**E47**	83	111.4	206	5.2
**E48**	92	119.6	179	5.2
**E49**	70	99.3	185	5.2
**E50**	61	98.8	207	5.1
**E51**	58	100.1	217	5.2
**E52A**	>15	-	366	5.1
**E53**	53	64.0	270	5.1
**E54A**	>15	-	344	5.1
**E56A**	>15	-	306	5.2
**E57**	55	85.9	252	5.4
**E58**	69	85.0	192	5.1
**E59**	24	35.3	373	5.2
**E60**	57	97.4	193	5.1
**E61**	39	82.7	262	5.3
**E62**	47	83.7	231	5.1
**E63**	85	92.4	204	5.3
**E64**	36	42.8	357	4.7
**E65**	125	130.6	160	5.1
**E66A**	>15	-	353	5.1
**E67A**	>13	-	288	5.1
**E68A**	>15	-	244	5.1
**E69**	27	55.6	345	5.1
**E72**	77	112.0	256	5.2
**E75**	33	75.8	196	5.1
**E77**	67	96.8	184	5.1
**E79**	42	38.2	891	5.5
**E80**	34	73.9	201	5.1
**E86**	29	50.3	333	5.2
**E87**	44	58.9	207	4.8
**E88**	37	91.0	175	4.9
**E89**	37	63.5	221	4.9
**E90**	36	98.3	176	5.1
**E91**	28	62.8	258	5.2

Table S4

Isolate	Average coverage (×)	N50 (Kb)	Number of contigs	Assembly size (Mb)
**E92**	33	58.9	188	4.9
**E93**	25	41.6	580	5.3
**E94**	49	119.6	159	5.2
**E95**	33	84.1	207	5.3
**E96**	119	68.3	229	5.1
**E97**	43	123.3	150	5.1
**E98**	113	120.2	173	5.1
**E99**	110	107.6	177	5.3
**E100**	128	129.9	185	5.5
**E101**	121	110.3	148	5.0
**E102**	105	111.4	186	5.1
**E104**	88	84.7	163	5.1
**E105**	43	95.7	189	5.1
**E106**	114	116.3	151	5.0
**E107**	74	97.6	186	5.2
**E109**	109	108.9	177	5.1
**E110**	95	112.3	226	5.1
**E111**	114	109.2	185	5.1
**E112**	85	96.6	143	5.0
**E113**	36	76.5	185	5.1
**E114**	116	102.3	159	5.1
**E116**	141	103.8	158	5.1
**E117**	72	75.0	146	4.7
**E118**	117	112.2	178	4.9
**E119**	111	94.0	121	4.9
**E120**	111	53.2	241	4.7
**E121**	73	92.2	168	5.0
**E122**	72	94.6	152	5.1
**E123**	116	90.0	178	5.0
**E124**	80	61.9	264	5.3
**E125**	100	128.1	163	5.2
**E126**	138	140.8	119	5.2
**E127**	76	97.2	161	5.1
**E128**	113	106.4	188	5.2
**E129**	78	137.6	121	5.2
